# Protective Role of Adenosine Triphosphate Against Tamoxifen-Induced Retinal Toxicity in a Rat Model

**DOI:** 10.3390/medicina62040787

**Published:** 2026-04-19

**Authors:** Ezgi Karatas, Bulent Yavuzer, Seher Koksaldi, Mustafa Kayabasi, Esra Tuba Sezgin, Cengiz Sarigul, Ozlem Demir, Bahadir Suleyman, Halis Suleyman

**Affiliations:** 1 Department of Ophthalmology, Faculty of Medicine, Agri Ibrahim Cecen University, Agri 04200, Turkey; ezkaratas@agri.edu.tr (E.K.); skayabasi@agri.edu.tr (S.K.); 2 Department of Pharmacology, Faculty of Medicine, Erzincan Binali Yıldırım University, Erzincan 24100, Turkey; bulent.yavuzer@erzincan.edu.tr (B.Y.); bsuleyman@erzincan.edu.tr (B.S.); 3 Department of Ophthalmology, Mus State Hospital, Mus 49200, Turkey; mkayabasi94@gmail.com; 4 Anesthesia Program, Vocational School of Health Services, Erzincan Binali Yıldırım University, Erzincan 24036, Turkey; esra.demir@erzincan.edu.tr; 5 Department of Medical Biochemistry, Faculty of Medicine, Erzincan Binali Yıldırım University, Erzincan 24100, Turkey; cengiz.sarigul@erzincan.edu.tr; 6 Department of Histology, Faculty of Medicine, Erzincan Binali Yıldırım University, Erzincan 24100, Turkey; ozlem.abuc@erzincan.edu.tr

**Keywords:** 8-hydroxy-2′-deoxyguanosine, adenosine triphosphate, DNA damage, drug-related side effects and adverse reactions, oxidative stress, rats, retinal diseases, retinal toxicity, tamoxifen

## Abstract

*Background and Objectives*: Tamoxifen, a cornerstone selective estrogen receptor modulator in breast cancer therapy, is increasingly recognized to be associated with retinal toxicity characterized by mitochondrial dysfunction, oxidative stress, lipid peroxidation, and oxidative DNA injury. By targeting mitochondrial bioenergetic dysfunction and redox disequilibrium, adenosine triphosphate (ATP) emerges as a biologically plausible candidate for retinal cytoprotection. This study aimed to evaluate the protective effect of ATP against tamoxifen-induced retinal toxicity in a rat model. *Materials and Methods*: Twenty-four male albino Wistar rats were randomly assigned to four groups: healthy control (HG), ATP-alone (ATPG, 4 mg/kg, intraperitoneally), tamoxifen-alone (TAMG, 5 mg/kg, orally), and tamoxifen plus ATP-treated (ATAG; ATP, 4 mg/kg, intraperitoneally; tamoxifen, 5 mg/kg, orally). Treatments were administered once daily for 30 days. Oxidative stress markers (malondialdehyde, total glutathione), antioxidant enzyme activities (superoxide dismutase, catalase), and oxidative DNA damage (8-hydroxy-2′-deoxyguanosine) were assessed in ocular tissues. Retinal histopathological evaluation included hematoxylin–eosin staining with semiquantitative assessment of edema, vascular congestion, polymorphonuclear leukocyte infiltration, and cytoplasmic vacuolization, together with quantitative measurements of retinal layer thicknesses and ganglion cell layer (GCL) cell counts. *Results*: Tamoxifen administration induced marked oxidative stress, antioxidant depletion, and increased oxidative DNA damage in ocular tissues, accompanied by significant thickening of retinal layers, reduced GCL cell counts, and pronounced disruption of retinal architecture. By comparison, ATP co-administration significantly suppressed lipid peroxidation and restored antioxidant defenses, thereby reducing oxidative DNA damage and preserving retinal structural integrity, as reflected by partial normalization of retinal layer thicknesses, preservation of GCL cell counts, and the presence of only mild residual edema. *Conclusions*: These findings indicate that ATP attenuates tamoxifen-induced retinal toxicity by supporting mitochondrial energy balance and redox homeostasis. Accordingly, ATP administration may represent a promising protective approach for reducing retinal injury associated with long-term tamoxifen therapy.

## 1. Introduction

Tamoxifen is a selective estrogen receptor modulator employed in the treatment of breast cancer in both males and females [1]. Tamoxifen is well established as an effective therapeutic agent for the treatment of patients with estrogen receptor (ER)-positive tumors [2]. This antitumor effect of tamoxifen is thought to be mediated predominantly through its anti-estrogenic effects. This anti-estrogenic effect is attributed to its competitive inhibition of estrogen binding to estrogen receptors [3]. Tamoxifen has been demonstrated to induce apoptosis not only in ER-positive breast cancer cells but also in ER-negative cells [4]. However, similar to many anticancer agents, tamoxifen can lead to severe and even life-threatening adverse effects [1]. Given the presence of estrogen receptors in the retina, retinal pigment epithelium, and choroid, these tissues are likewise susceptible to the effects of tamoxifen [5]. Case reports have described crystalline retinal deposits, macular edema, and corneal alterations as potential manifestations of tamoxifen-induced ocular toxicity [6]. A case report documented a characteristic presentation of tamoxifen retinopathy with concurrent moderate uveitis, observed even at low doses (<30 mg/day) [7]. The literature emphasizes that tamoxifen-induced retinopathy is an irreversible condition [8]. Tamoxifen, along with many other drugs, has been reported to induce retinopathies through mechanisms involving ocular oxidative stress [9]. Recent experimental evidence further suggests that impaired antioxidant defense pathways may increase oxidative vulnerability in ocular tissues. In particular, downregulation of GCLM/LANCL1 has been shown to enhance oxidative stress susceptibility in ocular epithelial cells, highlighting the role of disrupted antioxidant homeostasis in retinal injury [10]. The available report indicates that tamoxifen increases intramitochondrial ionized Ca^2+^ concentrations in rat liver and human breast cancer MCF-7 cells, while concurrently inducing mitochondrial lipid peroxidation (LPO) [11]. The literature further indicates that tamoxifen suppresses mitochondrial membrane potential and respiration, substantially reduces adenosine triphosphate (ATP) levels, and induces mitochondrial LPO [12]. Additionally, it has been reported that microsomal activation of tamoxifen generates reactive oxygen species (ROS), thereby leading to the formation of 8-hydroxy-2′-deoxyguanosine (8-OHdG) [13]. As is well established, 8-OHdG is a product of DNA oxidation and is widely utilized as a biomarker of oxidative stress [14].

Notably, the presence of hyperlipidemia has been shown to be significantly associated with tamoxifen retinopathy; because tamoxifen forms drug–lipid complexes that impede the normal lysosomal catabolism of lipids, hyperlipidemia increases the risk of tamoxifen-induced toxicity in these patients [15]. ATP production occurs under both aerobic and anaerobic conditions through processes such as cellular respiration, β-oxidation, ketosis, and the catabolism of lipids and proteins [16]. ATP is utilized as an energy source in numerous cellular processes, including ion transport, muscle contraction, neural signal transmission, antioxidant defense against ROS, and the synthesis of various biochemical compounds [16,17].

Taken together, tamoxifen inhibits normal lipid catabolism, promotes hyperlipidemia, reduces intracellular ATP levels, and induces oxidative stress; moreover, the impairment of lipid catabolism further exacerbates the decline in ATP production. The selected doses in the present study were determined based on well-established experimental models reported in the literature, in which oral tamoxifen at 5 mg/kg has been used to induce oxidative stress-related tissue injury, whereas intraperitoneal ATP at 4 mg/kg has been shown to exert protective effects in experimental ocular injury models [18,19,20]. These studies demonstrate that ATP may treat tamoxifen-associated retinopathy. ATP has not been shown to protect against tamoxifen-induced retinopathy. In a rat model, this study examined whether ATP could protect the retina from tamoxifen.

## 2. Materials and Methods

### 2.1. Animals

Twenty-four male rats of the Wistar albino strain, verified at 9–10 weeks of age and weighing 262–275 g at baseline, were enrolled in the study to preserve a uniform experimental population and to minimize potential confounders associated with age- or weight-related physiological differences. All rats were sourced from the Erzincan Binali Yıldırım University’s experimental animal facility (Erzincan, Turkey). The rats were randomly assigned to four experimental groups (*n* = 6 per group), and care was taken to preserve comparable baseline body weight profiles across groups to reduce potential sources of confounding. Before the experimental procedures commenced, the animals underwent a one-week acclimatization period during which they were housed in standard wire-mesh cages (20 cm × 35 cm × 55 cm; 1925 cm^2^ floor area), each accommodating six rats. Environmental parameters were tightly regulated, including a 12 h light/12 h dark cycle with environmental temperature set at 22 ± 2 °C, and humidity levels kept within 30–70%. Throughout the study period, the animals had unrestricted access to tap water and standard commercial rodent chow (Bayramoglu Feed and Flour Industry Inc., Erzurum, Turkey).

All experimental manipulations were performed within the accredited research laboratories of the Experimental Animals Application and Research Center of Erzincan Binali Yıldırım University, ensuring adherence to institutional and regulatory standards. The experimental protocol was developed and executed in compliance with the European Parliament Directive 2010/63/EU regarding the safeguarding of animals utilized in experimental studies (Ethics approval ID: 2016-24-199). All scientific techniques and reporting standards adhered to the ARRIVE (Animal Research: Reporting of In Vivo Experiments) criteria [21].

### 2.2. Chemicals and Reagents

All chemical agents employed in the study were of analytical grade and procured from certified suppliers. Thiopental sodium (Pental Sodyum^®^, 0.5 g vial; Catalog No.: 8699508270385) was obtained from Menarini Health and Pharmaceuticals Industry Trade Inc. (Istanbul, Turkey). Tamoxifen citrate (Tamoxifen^®^, 10 mg tablet; Catalog No.: 8699525019783) was supplied by Deva Holding Inc. (Istanbul, Turkey). Adenosine triphosphate (ATP^®^, 10 mg/mL injectable solution; Catalog No.: 4820117741513) was purchased from Zdorovye Narodu Pharmaceutical LLC. (Kharkiv, Ukraine). Tamoxifen tablets were freshly crushed using a mortar and pestle and suspended in distilled water immediately prior to administration to minimize potential variability associated with tablet excipients and to ensure dosing accuracy. The required dose was calculated individually based on body weight (mg/kg), and the corresponding amount of powdered tablet was precisely weighed using a calibrated analytical balance. The resulting suspension was thoroughly homogenized prior to each administration and delivered by oral gavage. ATP was prepared from a sterile injectable solution (10 mg/mL) and administered intraperitoneally without further dilution immediately prior to injection to preserve its biochemical stability.

### 2.3. Experimental Design and Randomization

Animal numbers were determined based on the requirement to balance ethical responsibility with scientific rigor, employing only the smallest group size capable of producing dependable and replicable outcomes in line with the 4R (Reduction, Refinement, Replacement, and Responsibility) framework [22]. The number of subjects was determined based on with reference to previously published experimental studies investigating oxidative stress–mediated retinal or optic nerve injury in rat models employing comparable biochemical and histopathological endpoints. In these studies, group sizes of approximately six animals were commonly adopted and demonstrated sufficient statistical sensitivity to detect significant alterations in oxidative stress parameters and tissue morphology [18,20,23]. Accordingly, a sample size of six animals per group was considered methodologically appropriate for the present experimental design. Furthermore, for retinal morphometric evaluation, multiple microscopic fields obtained from each section were systematically analyzed in order to enhance measurement robustness and minimize sampling variability. This approach is consistent with established practices in preclinical ophthalmic research and aligns with current recommendations for achieving an optimal balance between statistical validity and ethical responsibility. Each stage of the experimental procedure included its own set of exclusion criteria. Prior to the experimental procedures, the subjects displaying aberrant posture, diminished spontaneous activity, or injuries from aggressive contacts with cage mates were eliminated before random allocation and initiation of the experimental procedures. The peri- and post-experimental phases included exclusion criteria that addressed issues potentially compromising data integrity or animal welfare. The criteria encompassed unexpected mortality or complications related to anesthesia or drugs occurring prior to the designated predefined outcomes; administration-related errors, including unsuccessful intragastric administration or leakage at the injection site; deviations from the planned treatment regimen protocol or incomplete administration of the experimental compounds; significant weight loss surpassing 15–20% of baseline, dehydration, or other evidence of systemic illness; pronounced signs of distress, including self-harm or prolonged vocalization indicative of uncontrolled pain; inability to complete behavioral assessments due to lack of compliance or motor impairments unrelated to the intervention; and compromised tissue integrity occurring during sample collection or processing that could compromise reliable histological or biochemical evaluation. The exclusion criteria were strictly enforced during the intervention period and the subsequent assessment of experimental data. All animals satisfied the established inclusion criteria throughout the pre-experimental, peri-experimental, and post-experimental periods, thereby remaining in the study. Group allocation was conducted utilizing a random-number system to minimize allocation bias among groups to the experimental groups. To minimize potential sources of confounding and bias, each cage and animal was assigned a unique numerical identifier that remained constant throughout the trial.

### 2.4. Experimental Groups

The experimental design included four groups: a healthy control group (HG); an ATP-treated group receiving 4 mg/kg ATP intraperitoneally (ATPG); a tamoxifen-treated group administered 5 mg/kg tamoxifen orally (TAMG); and a combined treatment group receiving 4 mg/kg ATP intraperitoneally along with 5 mg/kg tamoxifen orally (ATAG).

### 2.5. Experimental Procedure

ATP was administered intraperitoneally at a dose of 4 mg/kg to the ATPG (*n* = 6) and ATAG (*n* = 6) groups [18]. The animals enrolled in the TAMG (*n* = 6) and HG (*n* = 6) groups were administered distilled water as the vehicle control through the same route of administration. One hour after ATP or vehicle delivery, tamoxifen was administered orally at a dose of 5 mg/kg to the TAMG and ATAG groups [19]. This dosing regimen was applied once daily for a duration of one month. The selected doses were determined based on well-characterized experimental paradigms reported in the literature. Intraperitoneal administration of ATP at 4 mg/kg has been shown to exert protective effects in models of drug-induced retinal and optic nerve injury [18,20], whereas oral tamoxifen at 5 mg/kg is widely used in rodent models to induce oxidative stress–mediated tissue damage [19]. The chosen dose regimen was predicated on prior experimental investigations that assessed the protective benefits of ATP in models of drug-induced ocular or brain tissue harm. In these studies, ATP was administered as a pretreatment to allow systemic exposure before the toxic insult [18,20]. Therefore, tamoxifen was administered orally one hour after ATP administration in order to evaluate the potential protective effects of ATP against tamoxifen-induced retinal injury. Purinergic signaling is known to play important physiological and pathological roles in ocular tissues, including retinal neurons and glial cells [24]. However, ocular pharmacokinetics of systemically administered ATP were not directly assessed in the present study; therefore, the timing of administration was determined based on previously reported experimental protocols. At the conclusion of the treatment period, animals were euthanized under high-dose anesthesia (50 mg/kg thiopental sodium). Following euthanasia, the globes were enucleated, and oxidative–antioxidant biochemical parameters were quantified in the ocular tissues. Retinal tissues also underwent histopathological evaluation. The experimental data obtained from all groups were subsequently analyzed and compared to characterize intergroup differences.

### 2.6. Biochemical Analyses

#### 2.6.1. Preparation of Tissue Samples for MDA, tGSH, SOD, and CAT Analyses

The animal’s ocular tissues were carefully removed and then washed with ice-cold isotonic saline solution to remove circulating blood and surface debris. Approximately 70 mg of tissue from each rat was weighed and promptly processed by mincing into small fragments, followed by quick snap-freezing in liquid nitrogen. The frozen tissue was then ground into a uniform, fine powder using a pre-cooled mortar and pestle to preserve molecular integrity. The powdered tissue was then homogenized in phosphate-buffered saline (PBS, pH 7.4) at a standardized 1:10 (*w*/*v*) ratio to optimize the extraction of biological components. The homogenized samples were vortexed for 10 s and then centrifuged at 10,000× *g* for 20 min at 4 °C to obtain clarity. The supernatant was cautiously collected and preserved at −80 °C until biochemical assays were conducted. To ensure uniformity in data interpretation and facilitate meaningful intergroup comparisons, all biochemical measures were corrected for total protein levels. MDA and tGSH levels were quantified as nmol per mg of protein, while SOD and CAT activity were reported as units per mg of protein.

#### 2.6.2. Quantification of MDA, tGSH, SOD, CAT, and Total Protein in Ocular Tissue

MDA and tGSH content, together with SOD activity, quantification in ocular tissue samples was performed using rat-specific ELISA kits (BT Lab, Shanghai Korain Biotech Co., Ltd., Shanghai, China; Catalog Nos. E0156Ra for MDA, E1101Ra for tGSH, and E0168Ra for SOD), and CAT activity was determined using a rat-specific ELISA kit (REL ASSAY Diagnostics, Gaziantep, Turkey; Catalog No. RL0253), according to the respective manufacturers’ instructions. All ELISA measurements were performed according to the manufacturers’ validated protocols. The analytical sensitivity and the intra- and inter-assay coefficients of variation were within the ranges specified in the manufacturers’ technical documentation. Tissue homogenates were prepared and diluted according to the recommended procedures in order to minimize potential matrix effects associated with ocular tissue samples. Total protein content was determined using the Bradford assay [25], using a Coomassie Brilliant Blue G-250–based colorimetric method (Cat. No. 115444, Sigma-Aldrich Chemie GmbH, Taufkirchen, Germany), dye to protein molecules and the associated absorbance shift. Spectrophotometric measurements were taken at 595 nm, and the resulting protein concentrations were utilized to standardize all biochemical parameters.

#### 2.6.3. Preparation of Tissue Samples for 8-OHdG Analysis

To determine DNA oxidation levels, genomic DNA was isolated from tissue samples. Briefly, 50 mg of tissue was homogenized in 1 mL of ice-cold homogenization buffer (30 mM Tris–Hydrochloric acid, pH 8.0; 10 mM EDTA; 10 mM 2-mercaptoethanol; 0.5% (*v*/*v*) Triton X-100; Sigma-Aldrich (Merck KGaA, Darmstadt, Germany) at 4 °C. The resulting homogenate was centrifuged at 1000× *g* for 10 min, and the supernatant was discarded. The obtained pellet was resuspended in 1 mL of extraction buffer (0.1 M Tris–Hydrochloric acid, pH 8.0; 0.1 M NaCl; 20 mM EDTA) and mixed thoroughly by vortexing for 30 s. The suspension was then centrifuged at 1000× *g* for 2 min. The pellet was again resuspended in the same extraction buffer and thoroughly mixed by vortexing. Subsequently, 400 µL of phenol (Sigma-Aldrich [Merck KGaA, Darmstadt, Germany]) was added to the suspension, followed by vortexing for 1 min. After allowing phase separation for 10 min, the upper aqueous phase was carefully transferred to a clean tube. Next, 400 µL of chloroform–isopropanol (24:1, *v*/*v*; Sigma-Aldrich [Merck KGaA, Darmstadt, Germany]) was added, and the mixture was centrifuged at 10,000× *g* for 10 min. The resulting upper phase was transferred to a new tube. For DNA precipitation, 40 µL of 3 M sodium acetate (pH 5.0; Merck KGaA, Darmstadt, Germany) and 800 µL of cold ethanol (Merck KGaA, Darmstadt, Germany) were added and gently mixed. The samples were centrifuged at 10,000× *g* for 15 min, after which the supernatant was completely removed. The DNA pellet was washed with 1 mL of 70% ethanol. For DNA hydrolysis, 1 mL of the obtained DNA solution was mixed with 0.5 mL of 60% formic acid (Sigma-Aldrich [Merck KGaA, Darmstadt, Germany]) and incubated at 150 °C for 60 min. Following incubation, the tubes were allowed to equilibrate at room temperature to facilitate formic acid evaporation. Approximately 1 mL of the resulting hydrolysate was stored at −20 °C until analysis.

#### 2.6.4. Biochemical Determination of the DNA Oxidation Product 8-OHdG in Ocular Tissue

The levels of 8-hydroxy-2′-deoxyguanosine (8-OHdG), a biomarker of oxidative DNA damage, and deoxyguanosine (dG) were determined using high-performance liquid chromatography (HPLC). dG concentrations were quantified using an ultraviolet (UV) detector, whereas 8-OHdG levels were measured using an electrochemical detection (ECD) system. Prior to HPLC analysis, hydrolyzed DNA samples were reconstituted in the HPLC mobile phase, and the final volume was adjusted to 1 mL. An aliquot of 20 µL from each sample was injected into the HPLC-ECD system (Agilent 1100 modular system equipped with an HP 1049A electrochemical detector; Agilent Technologies, Waldbronn, Germany). Chromatographic separation was performed using a reverse-phase C18 (RP-C18) analytical column (250 mm × 4.6 mm, particle size 4.0 µm; Phenomenex Inc., Torrance, CA, USA). The mobile phase consisted of 0.05 M potassium phosphate buffer (pH 5.5) containing 3% (*v*/*v*) acetonitrile (Merck KGaA, Darmstadt, Germany) and was delivered at a flow rate of 1.0 mL/min. dG was detected spectrophotometrically at a wavelength of 245 nm, whereas 8-OHdG was electrochemically detected at an applied potential of 600 mV. Quantification of dG and 8-OHdG was performed using commercially available analytical standards (Sigma-Aldrich [Merck KGaA, Darmstadt, Germany]), and their concentrations were initially determined in pmol/L. The extent of oxidative DNA damage was expressed as the ratio of 8-OHdG to deoxyguanosine (dG) and reported as the number of 8-OHdG molecules per 10^6^ dG.

### 2.7. Histological Processing and Analysis

All harvested ocular tissues were preserved in 10% formalin buffered to neutral pH for roughly three days to guarantee excellent cellular and structural morphology retention. Subsequent to fixation, the samples were placed in processing cassettes and subjected to continuous rinsing with tap water for twenty-four hours to eliminate residual formaldehyde. The tissues were further dehydrated using a graded sequence of ethanol comprising a graded ethanol series (70–100%) to remove water from the tissue environment. Subsequent to dehydration, the samples were clarified in xylene and embedded in paraffin to produce homogeneous blocks appropriate for microtomy. Tissues fixed in paraffin were sectioned at 4–5 µm utilizing a rotary microtome, and the resulting sections were subsequently stained with hematoxylin and eosin (H&E) [26]. Microscopic evaluation was performed with a light microscope (Olympus BX53; Olympus^®^ Inc., Tokyo, Japan) equipped with the DP2 SAL imaging system (version 3.3.1.198; Olympus^®^ Inc., Tokyo, Japan). Quantitative retinal morphometry was performed using the Image J (version 1.54i; National Institutes of Health, Bethesda, MD, USA) software platform and included measurements of the inner plexiform layer (IPL), the inner nuclear layer (INL), the outer nuclear layer (ONL), and total retinal thickness (TR), together with enumeration of ganglion cells within the ganglion cell layer (GCL). Histopathological alterations in the retinal tissue were evaluated according to the presence of edema, defined as reduced intercellular cohesion with expansion of the intercellular spaces; vascular congestion, characterized by increased vascular density and erythrocyte accumulation along the vessel walls; polymorphonuclear leukocyte infiltration, indicating the presence of inflammatory cell aggregates; and cytoplasmic vacuolization. From the serially obtained sections, six representative microscopic fields (one central and five peripheral) were examined in each section, with six sections evaluated per experimental group under 400× magnification. Semiquantitative scoring of tissue injury was applied using the following criteria: zero indicating an absence of damage, one indicating mild changes, two indicating moderate alterations, and three indicating severe injury [27]. All histopathological assessments were conducted by a seasoned histologist who remained unaware of the experimental group allocations during the assessment process.

### 2.8. Statistical Analysis

IBM SPSS^®^ Statistics for Windows (version 27.0; IBM Corp., Armonk, NY, USA, 2020) was used for all statistical analyses related to biochemical and histological data. GraphPad Prism^®^ (version 8.0.1; GraphPad Software, San Diego, CA, USA, 2018) was used to construct the graphical outputs. Measurements of retinal layer thickness and biochemistry are reported as mean ± standard error of the mean (SEM). The distributional characteristics of each biochemical and retinal layer thickness dataset were examined using the Shapiro–Wilk test to assess normality, while the homogeneity of variances was evaluated with Levene’s test (Tables S1–S4). Group differences were examined using a one-way analysis of variance (ANOVA) when both hypotheses (CAT, 8-OHdG) were satisfied. Tukey’s Honestly Significant Difference (HSD) test was then used for post hoc comparisons. Welch’s ANOVA was used in situations where the assumption of homogeneity was broken (MDA, tGSH, SOD), and the Games–Howell test was used for pairwise comparisons (Tables S5). For retinal layer thickness measurements (IPL, INL, ONL, and TR), Welch’s ANOVA followed by the Games–Howell post hoc test was applied (Table S6), as the assumptions of normality and/or homogeneity of variances were not consistently satisfied. Histopathological grading scores and number of ganglion cells in the GCL which represent ordinal data are reported as median values together with their minimum and maximum observations. For the histopathological grading scores and number of ganglion cells in the GCL dataset, the Kruskal–Wallis test, a nonparametric approach, was employed to determine overall group differences. Upon identifying significant differences, Dunn’s post hoc comparisons with Bonferroni correction were carried out to evaluate pairwise comparisons. A *p*-value < 0.05 was considered indicative of statistical significance.

## 3. Results

### 3.1. Biochemical Results

#### 3.1.1. Evaluation of Ocular Tissue MDA and tGSH Levels

MDA levels in the ATPG (4.44 ± 0.08) were slightly lower than those observed in the HG (4.54 ± 0.06); however, no statistically meaningful difference was identified (*p* = 0.740). In contrast, in TAMG (6.69 ± 0.06) showed a significant elevation in MDA levels compared with both the HG and the ATPG (*p* < 0.001). Co-administration of ATP (ATAG, 4.66 ± 0.15) significantly suppressed the tamoxifen-caused increase in MDA levels yielding values similar to the values in the HG (*p* = 0.888).

**Figure 1:**
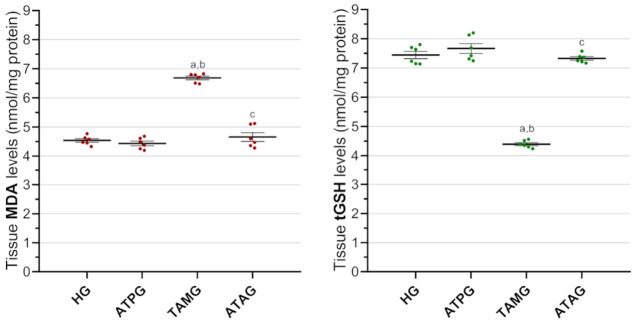
Effects of ATP and tamoxifen on MDA and tGSH levels in rat ocular tissue. Footnotes: Data are presented as mean ± SEM (standard error of the mean). Statistical significance was assessed by Welch’s ANOVA followed by the Games–Howell post hoc test. a, vs. HG, *p* < 0.001; b, vs. ATPG, *p* < 0.001; c, vs. TAMG, *p* < 0.001. For all groups, *n* = 6. Abbreviations: HG, healthy group; ATPG, ATP-alone group; TAMG, tamoxifen-alone group; ATAG, ATP + tamoxifen group; ATP, adenosine triphosphate; MDA, malondialdehyde; tGSH, total glutathione.

With respect to antioxidant status, ATP treatment alone (ATPG, 7.67 ± 0.17) induced a modest increase in tGSH levels relative to the HG (7.44 ± 0.12), although this difference was not statistically significant (*p* = 0.717). In contrast, TAMG (4.40 ± 0.05) showed a significant reduction in tGSH levels compared with both the HG and the ATPG (both *p* < 0.001). Notably, ATP co-administration (ATAG, 7.33 ± 0.06) markedly counteracted the tamoxifen-induced depletion of tGSH, restoring levels to values comparable to levels seen in the HG (*p* = 0.837; Figure 1).

#### 3.1.2. Analysis of Ocular Antioxidant Enzymes: SOD and CAT

ATP alone group (ATPG, 5.90 ± 0.13) exhibited a slight but non-significant elevation in ocular SOD activity versus the HG (5.73 ± 0.06; *p* = 0.654). In contrast, TAMG (3.34 ± 0.05) had a significant decrease in SOD activity compared to both HG and ATPG (*p* < 0.001). ATP co-administration (ATAG, 5.33 ± 0.09) significantly mitigated the tamoxifen-induced suppression of SOD activity (ATAG vs. TAMG, *p* < 0.001).

**Figure 2:**
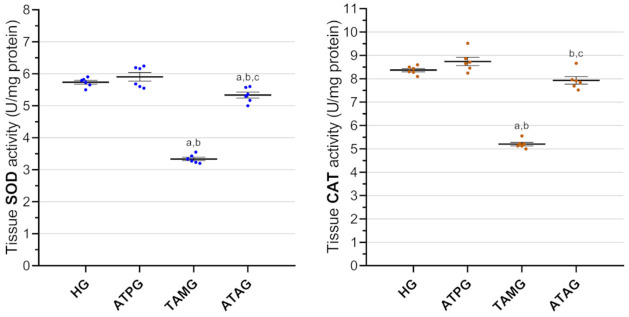
Effects of ATP and tamoxifen on SOD and CAT activities in rat ocular tissue. Footnotes: Data are presented as mean ± SEM (standard error of the mean). Statistical significance was assessed using Welch’s ANOVA followed by the Games–Howell post hoc test for SOD, and one-way ANOVA followed by Tukey’s HSD post hoc test for CAT. a, vs. HG, *p* < 0.05; b, vs. ATPG, *p* < 0.05; c, vs. TAMG, *p* < 0.001. For all groups, *n* = 6. Abbreviations: HG, healthy group; ATPG, ATP-alone group; TAMG, tamoxifen-alone group; ATAG, ATP + tamoxifen group; ATP, adenosine triphosphate; SOD, superoxide dismutase; CAT, catalase.

CAT levels in the HG (8.37 ± 0.07) were significantly higher than those observed in the TAMG (5.21 ± 0.08; *p* < 0.001), whereas no significant difference was detected between the HG and ATPG. ATP co-administration counteracted the tamoxifen-related reduction in CAT activity, resulting in values consistent with controls (*p* = 0.115; Figure 2).

#### 3.1.3. Analysis of Oxidative DNA Damage Marker 8-OHdG in Ocular Tissue

**Figure 3:**
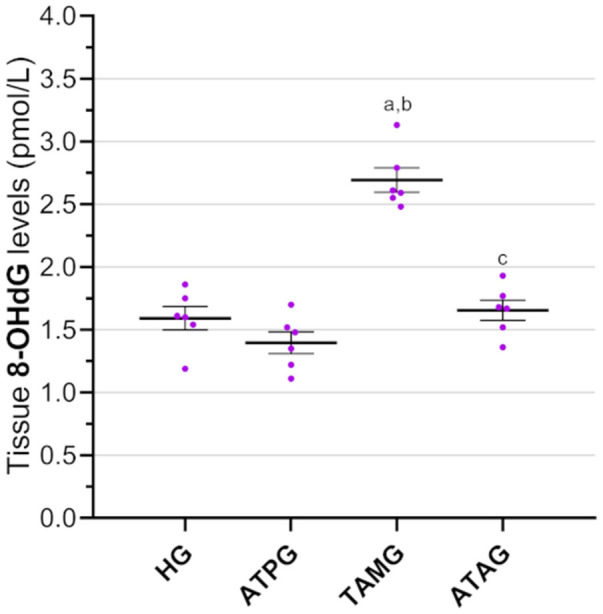
Effects of ATP and tamoxifen on 8-OHdG levels in rat ocular tissue. Footnotes: Data are presented as mean ± SEM (standard error of the mean). Statistical significance was assessed using one-way ANOVA followed by Tukey’s HSD post hoc test. a, vs. HG, *p* < 0.001; b, vs. ATPG, *p* < 0.001; c, vs. TAMG, *p* < 0.001. For all groups, *n* = 6. Abbreviations: HG, healthy group; ATPG, ATP-alone group; TAMG, tamoxifen-alone group; ATAG, ATP + tamoxifen group; ATP, adenosine triphosphate; 8-OHdG, 8-hydroxy-2′-deoxyguanosine.

There was no statistically significant difference in 8-OHdG levels between the ATPG (1.40 ± 0.09) and the HG (1.59 ± 0.09) (*p* = 0.437). In contrast, 8-OHdG levels were significantly higher in the TAMG (2.69 ± 0.10) compared with both the HG and ATPG (both *p* < 0.001), indicating increased oxidative DNA damage. ATP co-administration in ATAG had significantly reduced tamoxifen-induced 8-OHdG elevation (*p* < 0.001), with levels comparable to controls (*p* = 0.959) (Figure 3).

### 3.2. Histopathological Assessment

**Figure 4:**
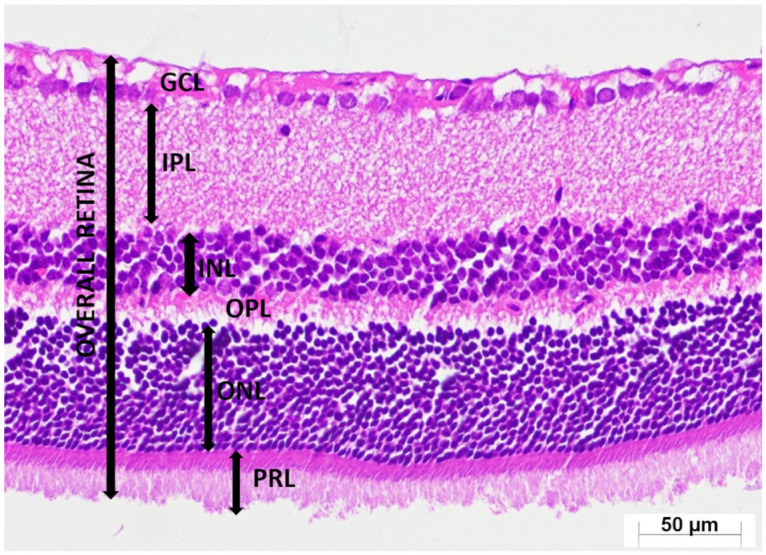
Representative photomicrograph of hematoxylin–eosin-stained retinal tissue from the healthy group (HG). Retinal layers are identified as the ganglion cell layer (GCL), inner plexiform layer (IPL), inner nuclear layer (INL), outer plexiform layer (OPL), outer nuclear layer (ONL), and photoreceptor layer (PRL). (H&E, ×400).

**Figure 5:**
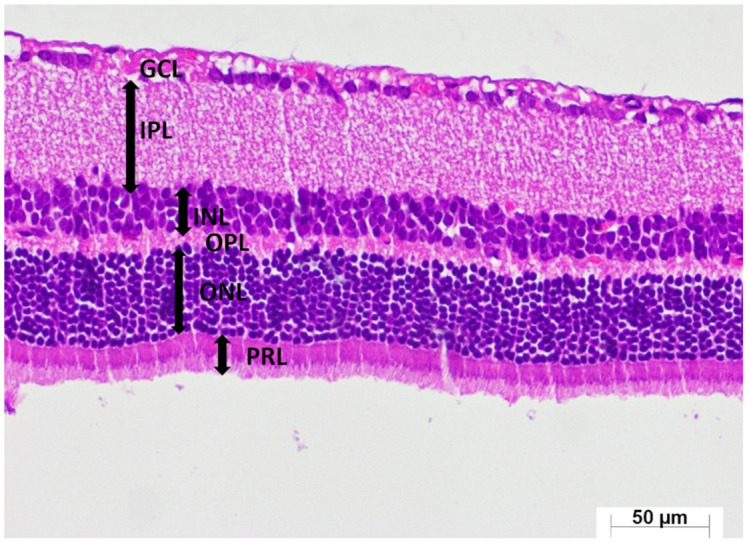
Representative photomicrograph of hematoxylin–eosin-stained retinal tissue from the ATP-alone group (ATPG). Retinal layers are identified as the ganglion cell layer (GCL), inner plexiform layer (IPL), inner nuclear layer (INL), outer plexiform layer (OPL), outer nuclear layer (ONL), and photoreceptor layer (PRL). (H&E, ×400).

**Figure 6:**
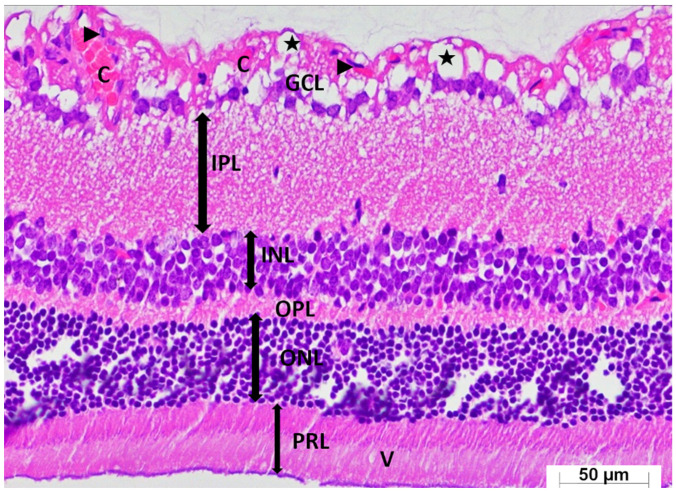
Representative photomicrograph of hematoxylin–eosin-stained retinal tissue from the tamoxifen-alone group (TAMG). Retinal layers are identified as the ganglion cell layer (GCL), inner plexiform layer (IPL), inner nuclear layer (INL), outer plexiform layer (OPL), outer nuclear layer (ONL), and photoreceptor layer (PRL). Arrowheads indicate polymorphonuclear cell infiltration; ★ denotes edema; C indicates dilated and congested capillary vessels; arrowheads indicate intercellular separation; and V denotes areas of vacuolization. (H&E, ×400).

**Figure 7:**
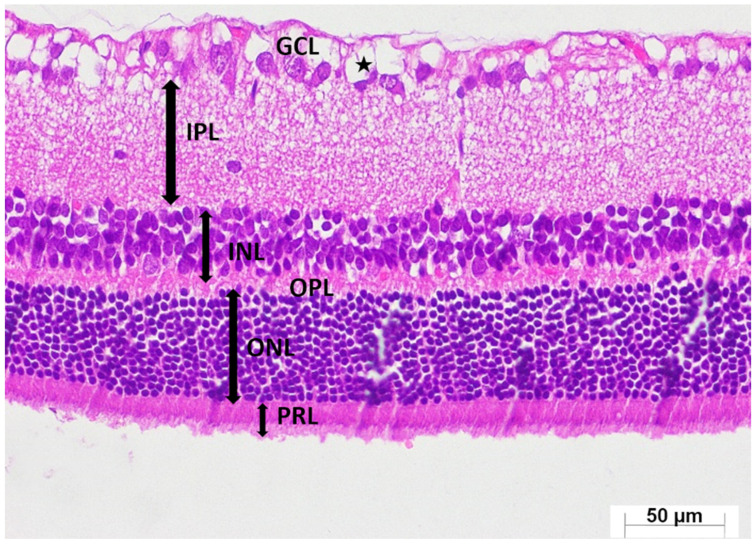
Representative photomicrograph of hematoxylin–eosin-stained retinal tissue from the ATP + tamoxifen group (ATAG). Retinal layers are identified as the ganglion cell layer (GCL), inner plexiform layer (IPL), inner nuclear layer (INL), outer plexiform layer (OPL), outer nuclear layer (ONL), and photoreceptor layer (PRL). ★ denotes edema. (H&E, ×400).

A thorough semi-quantitative assessment of retinal histopathological changes, retinal layer thickness measurements, and the number of ganglion cells in the GCL, along with the corresponding intergroup statistical analyses, letter-based group comparisons, and associated *p*-values, is presented in Table 1. Histological examination of retinal sections from the healthy group revealed a well-preserved retinal architecture, with the GCL, IPL, INL, OPL, ONL, and PRL exhibiting normal thickness and orderly structural organization. Cells within the INL and ONL displayed a uniform arrangement, and their nuclei were distinctly basophilic, indicative of preserved cytological integrity (HG; Figure 4). Retinal specimens obtained from animals receiving ATP alone demonstrated histomorphological features comparable to those of the healthy group; all retinal layers maintained normal thickness and cellular organization, with no observable pathological alterations (ATPG; Figure 5). In marked contrast, retinal sections from the tamoxifen-only group exhibited pronounced structural disruptions. A conspicuous thickening of the retinal layers was observed, along with a substantial reduction in the number of ganglion cells within the GCL. Within this layer, vascular elements demonstrated marked congestion and dilatation, accompanied by prominent interstitial edema and a pronounced infiltration of polymorphonuclear leukocytes. The ONL displayed considerable disorganization of intercellular connections, characterized by striking cellular dissociation, whereas the PRL contained distinct vacuolization areas indicative of tamoxifen-induced cytotoxic injury (TAMG; Figure 6). Histological analysis of retinal tissue from the ATP co-treatment group revealed a near-restoration of normal retinal morphology. The laminar architecture closely approximated that of healthy controls. Only mild edema was detected within the GCL, and the ganglion cells exhibited preserved structural characteristics. Cells within the INL and ONL remained basophilic and well organized, and importantly, no vacuolization was observed within the photoreceptor layer (ATAG; Figure 7).

## 4. Discussion

In this particular research, thorough biochemical evaluations and in-depth histopathology investigations were used to investigate the protective benefits of ATP against tamoxifen-related retinal damage. Our results indicate that tamoxifen elicits a marked increase in oxidative indices accompanied by a concomitant decline in antioxidant parameters. ROS, which play a fundamental role in cellular signaling and the maintenance of homeostasis, disrupt redox equilibrium by suppressing endogenous antioxidant defense mechanisms, thereby exacerbating oxidative stress [28]. When present at high concentrations, ROS likewise function as a detrimental source of oxidative stress within the retina [29]. ROS are predisposed to being generated within the retina owing to its cellular architecture and anatomical configuration [30]. Therefore, the pathophysiology of certain eye diseases is closely linked to ROS-mediated damage [31]. The elevated MDA levels observed in the ocular tissue of the tamoxifen-treated group represent a toxic end product of LPO, a process triggered by ROS generated in excess during oxidative stress and targeting cellular membranes [32]. For this reason, the measurement of MDA serves as a highly important biomarker for assessing ROS-induced damage in both in vivo and in vitro settings [33]. In addition to its established therapeutic efficacy in breast cancer, tamoxifen has been associated with several adverse effects, including retinal damage [5,8]. Accumulating evidence suggests that oxidative stress–related pathways play a critical role in tamoxifen-induced tissue toxicity [4,11,12]. Sajadimajd et al. demonstrated that tamoxifen-induced oxidative stress is characterized by a reduction in endogenous antioxidant defenses, including GSH, SOD, and CAT, accompanied by increased MDA levels [34]. Compared with the healthy and ATP-treated groups, MDA levels in ocular tissues were markedly elevated in tamoxifen-treated animals. The findings of the current study indicate that tamoxifen produces oxidative stress via increased lipid peroxidation, consistent with previously documented results [35]. As is well established, GSH, SOD, and CAT constitute major endogenous antioxidant defense mechanisms that protect cells against oxidative stress-induced damage [36]. Numerous studies have demonstrated the tissue-protective effects of antioxidant compounds against oxidative damage [37,38,39,40]. Parvez et al. demonstrated that certain antioxidant agents are effective in attenuating tamoxifen-induced toxicity while simultaneously enhancing its therapeutic efficacy [41]. Famurewa et al. reported that zinc alleviates oxidative damage by preventing tamoxifen-induced suppression of antioxidant enzyme activities, including SOD and CAT [42]. Clinical studies by Grzegorzewska et al. have indicated that increasing stress levels are associated with a decline in SOD activity [43]. In accordance with these reports, our data indicate that the concentrations of essential antioxidant defense components, such as tGSH, SOD, and CAT, were markedly diminished solely in the tamoxifen-treated group.

Oxidative DNA damage can promote the formation of DNA–protein cross-links, trigger structural breaks within the DNA helix, and induce chemical alterations in nucleobase architecture [44]. Mitochondrial DNA represents one of the most critical molecular targets of elevated oxidative stress. 8-OHdG, a well-recognized product of oxidative DNA damage, serves as a key indicator of the cytotoxic effects induced by increased oxidative stress [45]. Persistent oxidative DNA damage has emerged as a central determinant of irreversible cellular injury. Converging experimental and clinical evidence indicates that sustained accumulation of DNA oxidation products, particularly 8-OHdG, compromises DNA repair capacity, disrupts mitochondrial homeostasis, and drives progressive tissue degeneration. In the context of retinal pathology, these processes are likely to contribute to the enduring structural and functional alterations observed in drug-induced retinopathies [46]. The findings of the present study are concordant with the pioneering investigations conducted by Ye and Bodell, which established the mechanistic basis of tamoxifen-induced oxidative DNA damage [13]. Our results suggest that the significantly elevated 8-OHdG levels detected in the retinal tissues of animals treated with tamoxifen alone are closely associated with tamoxifen-induced oxidative stress and enhanced ROS generation within the retina.

The literature indicates that tamoxifen impairs mitochondrial membrane potential and inhibits mitochondrial respiration, resulting in a significant depletion of cellular ATP content [12]. Impaired ATP synthesis facilitates excessive mitochondrial ROS generation, leading to amplified oxidative stress and pronounced membrane LPO [47]. Previous studies have demonstrated that diminished mitochondrial ATP availability in retinal ganglion cells compromises the structural integrity and functional performance of photoreceptors [48]. The present study demonstrated that ATP administration alone did not induce any significant alterations in oxidant or antioxidant status in ocular tissues compared with healthy controls. Furthermore, ATP administration markedly suppressed the tamoxifen-induced elevation of oxidative markers and the concomitant depletion of antioxidant molecules in ocular tissues. Bouitbir et al. demonstrated that cytotoxic drug-induced mitochondrial ROS overproduction leads to impaired ATP synthesis, thereby promoting mitochondrial dysfunction and subsequent tissue injury, a mechanism that is concordant with the findings of the present study [49]. The protective effects of ATP observed in this study may be attributed, at least in part, to its role in purinergic signaling and mitochondrial bioenergetics. Extracellular ATP acts as a signaling molecule through P2X and P2Y purinergic receptors, which are expressed in various retinal cells including neurons, glial cells, and vascular endothelium. Activation of these receptors has been shown to regulate inflammatory responses, modulate microglial activation, and promote neuronal survival under stress conditions [50,51]. In addition to its signaling role, ATP is essential for mitochondrial function and cellular energy homeostasis. Adequate ATP levels are critical for maintaining ion gradients, reducing oxidative stress, and preventing apoptotic cell death. Previous studies have demonstrated that mitochondrial dysfunction and ATP depletion contribute to retinal degeneration and drug-induced retinal toxicity [52,53]. Therefore, exogenous ATP administration may exert protective effects by supporting mitochondrial bioenergetics and enhancing cellular resilience against toxic injury. Yıldırım et al. reported that ATP administration mitigates anticancer drug-induced oxidative stress through the suppression of oxidant parameters and the augmentation of antioxidant molecular levels [23]. Accumulating evidence in the literature indicates that ATP plays a critical role in preserving cellular homeostasis [24]. In accordance with the literature, ATP markedly suppressed the tamoxifen-induced increase in MDA levels in ocular tissues and prevented the reductions in tGSH, SOD, and CAT levels.

The histopathological alterations observed in the present study closely paralleled the biochemical outcomes. A pronounced increase in retinal layer thickness was observed in the tamoxifen-treated group. A reduction in the number of cells within the GCL was observed, accompanied by vascular congestion and dilatation in the associated blood vessels. Additionally, marked edema and polymorphonuclear cell infiltration were evident in this stratum. The present results indicate that tamoxifen induces inflammatory responses via oxidative injury-related pathways. Previous studies supporting the present findings have demonstrated that tamoxifen induces degenerative alterations in the histological architecture of retinal nerves [54,55]. Histopathological assessment of retinal tissues from animals receiving combined ATP and tamoxifen treatment showed intact vascular and connective tissue structures as well as preserved astrocytic morphology, with only mild edema detected in the GCL. The current results align with the findings of Bayrakceken et al., who shown in a rat model of optic nerve damage that ATP administration mitigated degenerative and vacuolar alterations while inhibiting connective tissue development and astrocytic proliferation [20]. Given that the histopathological findings of the aforementioned studies demonstrating the protective effects of ATP on retinal tissue are consistent with the results of the present study, ATP may be considered a promising therapeutic candidate for the treatment of retinal injury [18]. Although the present study demonstrated significant biochemical and histopathological improvements following ATP administration, it should be noted that structural preservation does not necessarily indicate functional recovery of the retina. Electrophysiological techniques such as electroretinography (ERG) provide objective information about retinal function by measuring the electrical responses of photoreceptors and inner retinal neurons, and are widely used in experimental and clinical studies evaluating retinal toxicity and neuroprotection. ERG recordings can detect functional alterations that may not be evident from morphological assessment alone [56,57]. In addition to electrophysiological testing, in vivo imaging techniques such as optical coherence tomography (OCT) allow detailed visualization of retinal layer integrity and enable longitudinal monitoring of retinal structural changes in experimental models and human retinal diseases [58,59]. Importantly, several studies have demonstrated that combining ERG and OCT provides complementary information, allowing simultaneous evaluation of retinal structure and function and improving the interpretation of therapeutic effects in retinal injury models [60]. Therefore, although our findings indicate that ATP administration attenuates tamoxifen-induced biochemical and histopathological retinal damage, future studies incorporating functional assessments such as ERG together with in vivo structural imaging using OCT will be necessary to determine whether the observed structural protection translates into meaningful functional preservation of retinal activity.

## 5. Conclusions

The present study demonstrates that tamoxifen promotes a state of oxidative stress that contributes to the development of retinal injury. In contrast, ATP administration markedly attenuated tamoxifen-associated retinal toxicity. Collectively, these findings suggest that ATP supplementation may represent a potential prophylactic strategy in therapeutic settings involving chemotherapeutic agents with established cytotoxic liability, such as tamoxifen. However, comprehensive experimental investigations and well-designed clinical studies are required to substantiate this hypothesis and to further define the translational and therapeutic relevance of ATP in the prevention of drug-induced retinal damage.

### Limitations and Future Perspectives

Several limitations of the present study should be acknowledged. First, the experimental design was conducted exclusively in male albino Wistar rats, which restricts the evaluation of potential sex-specific differences in tamoxifen-induced retinal toxicity and ATP-mediated protection. Considering the established influence of sex hormones on oxidative stress regulation, mitochondrial function, and retinal vulnerability, future investigations incorporating female animal models are warranted to improve the generalizability and translational relevance of these findings. Second, the effects of tamoxifen and ATP were examined using single fixed doses administered over a defined experimental period. Although this approach facilitated mechanistic interpretation, it did not allow assessment of dose–response relationships, toxicity thresholds, or the temporal dynamics of retinal injury and recovery. Accordingly, future studies employing multiple dosing regimens, extended treatment durations, and delayed intervention protocols are necessary to more comprehensively characterize both the toxicodynamic profile of tamoxifen and the protective efficacy of ATP. Third, the current experimental design utilized animals free of pre-existing metabolic or systemic disease. In clinical practice, tamoxifen-associated retinal toxicity frequently develops in patients with concomitant metabolic disorders, including diabetes mellitus, hepatic dysfunction, and systemic oxidative burden, all of which may potentiate mitochondrial impairment and retinal susceptibility. Therefore, the absence of disease-specific experimental models represents an important limitation, and subsequent studies incorporating metabolically compromised or systemic pathology models may provide deeper insight into the protective capacity of ATP under clinically relevant conditions. Fourth, the present investigation primarily relied on biochemical and histopathological assessments to evaluate retinal injury. While these endpoints provide robust molecular and structural evidence, the lack of functional and in vivo imaging modalities, such as electroretinography (ERG) and optical coherence tomography (OCT), limits evaluation of visual function and retinal integrity in real time. Integration of such functional outcomes would substantially strengthen the translational value of future investigations. Fifth, although the protective effects of ATP against tamoxifen-induced retinal toxicity were primarily interpreted in relation to oxidative stress modulation, direct assessments of mitochondrial structure and function were not performed. Specifically, key parameters such as retinal ATP content, mitochondrial membrane potential, respiratory chain activities, and mitochondria-specific oxidative stress markers were not evaluated. Therefore, future studies incorporating targeted mitochondrial functional analyses are warranted to more clearly elucidate the underlying mechanistic pathways. Finally, although the findings demonstrate a protective role of ATP against tamoxifen-induced retinal damage in an experimental setting, direct clinical extrapolation remains constrained by interspecies differences in retinal metabolism, pharmacokinetics, and mitochondrial regulation. Consequently, further mechanistic studies, long-term safety evaluations, and well-designed translational and clinical investigations are required to determine whether ATP supplementation may offer therapeutic or prophylactic benefit in patients receiving tamoxifen therapy.

## Figures and Tables

**Table 1:** Comparative analysis of histopathological parameters in rat retinal tissue among experimental groups.

Histopathological Parameters	Groups					
	HG	ATPG	TAMG	ATAG	F or H	*p*
Histopathological grading data						
Edema ^a^	0 (0–0)	0 (0–0)	3 (2–3) ^c,d^	1 (0–2) ^c,d,e^	119.186 ^f^	<0.001
Vascular Congestion ^a^	0 (0–0)	0 (0–0)	3 (2–3) ^c,d^	0 (0–1) ^e^	123.702 ^f^	<0.001
PNL cell infiltration ^a^	0 (0–0)	0 (0–0)	2 (1–3) ^c,d^	0 (0–1) ^e^	126.102 ^f^	<0.001
Vacuolization ^a^	0 (0–0)	0 (0–0)	2 (1–3) ^c,d^	0 (0–1) ^e^	117.219 ^f^	<0.001
Retina thickness of layers (µm)						
Inner plexiform layer ^b^	40.34 ± 0.25	39.85 ± 0.27	63.81 ± 0.22 ^c,d^	40.74 ± 0.16 ^d,e^	2902.127 ^g^	<0.001 ^h^
Inner nuclear layer ^b^	21.91 ± 0.10	21.60 ± 0.13	33.00 ± 0.17 ^c,d^	22.99 ± 0.12 ^c,d,e^	1229.826 ^g^	<0.001 ^h^
Outer nuclear layer ^b^	45.95 ± 0.12	45.61 ± 0.14	55.97 ± 0.20 ^c,d^	46.83 ± 0.26 ^c,d,e^	703.411 ^g^	<0.001 ^h^
Total retina ^b^	152.39 ± 0.15	152.00 ± 0.13	205.84 ± 0.33 ^c,d^	152.87 ± 0.28 ^d,e^	7789.442 ^g^	<0.001 ^h^
Number of ganglion cells in the GCL ^a^	8 (7–9)	7 (7–9)	5 (4–6) ^c,d^	7 (6–8) ^c,e^	98.595 ^f^	<0.001

Footnotes: ^a^ indicates Kruskal–Wallis test, Dunn’s test with Bonferroni correction, median (maximum–minimum); ^b^ indicates Welch’s ANOVA, Games–Howell test, µm, mean ± standard error of the mean (SEM); ^c^ indicates *p* < 0.05 vs. HG, ^d^ indicates *p* < 0.05 vs. ATPG; ^e^ indicates *p* < 0.001 vs. TAMG; ^f^ indicates the test statistic is adjusted for ties; ^g^ indicates asymptotically F distributed; ^h^ indicates Welch’s ANOVA *p*-values. For all groups, *n* = 36. Abbreviations: HG, healthy group; ATPG, ATP-alone group; TAMG, tamoxifen-alone group; ATAG, ATP + tamoxifen group; ATP, Adenosine triphosphate; PNL, Polymorphonuclear leukocyte; GCL, ganglion cell layer.

## Data Availability

The original contributions presented in this study are included in the article/Supplementary Materials. Further inquiries can be directed to the corresponding author.

## Supplementary Materials

The following supporting information can be downloaded at: https://www.mdpi.com/article/10.3390/medicina62040787/s1, Table S1: Shapiro–Wilk normality test results for biochemical parameters in rat ocular tissue; Table S2: Levene’s test results for homogeneity of variances of biochemical parameters in rats; Table S3: Assessment of the normality of retinal layer thickness measurements in rat retina using the Shapiro–Wilk test; Table S4: Levene’s test results assessing the homogeneity of variances for retinal layer thicknesses; Table S5: Post hoc multiple comparisons of *p*-values reflecting the effects of ATP and tamoxifen on oxidant, antioxidant, and oxidative DNA damage biomarkers in rat ocular tissue; Table S6: Post hoc multiple comparisons of retinal layer thickness measurements among experimental groups following ATP and tamoxifen administration.
