# Supplementary material for: Protective Role of Adenosine Triphosphate Against Tamoxifen-Induced Retinal Toxicity in a Rat Model
Source: Medicina (Kaunas). 2026 Apr 19;62(4):787. doi: 10.3390/medicina62040787 (PMC13117042; doi:10.3390/medicina62040787)
Supplement: Supplementary file 1 [file medicina-62-00787-s001.zip › Table S1-R1.pdf]

Table S1. Shapiro–Wilk normality test results for biochemical parameters in rat ocular tissue

		Shapiro–Wilk	Biochemical Variables				
			MDA	tGSH	SOD	CAT	8-OHdG
Groups	HG	Statistic	0.984	0.830	0.963	0.974	0.921
		df	6	6	6	6	6
		Sig.	0.969	0.109	0.845	0.921	0.509
	ATPG	Statistic	0.945	0.868	0.804	0.921	0.982
		df	6	6	6	6	6
		Sig.	0.699	0.220	0.064	0.515	0.962
	TAMG	Statistic	0.837	0.949	0.931	0.868	0.836
		df	6	6	6	6	6
		Sig.	0.122	0.729	0.585	0.218	0.121
	ATAG	Statistic	0.849	0.924	0.950	0.865	0.982
		df	6	6	6	6	6
		Sig.	0.154	0.537	0.740	0.206	0.961

Footnotes: Normality assumptions were satisfied for all biochemical parameters across all experimental groups; therefore, between-group comparisons were conducted using analysis of variance (ANOVA). For all groups, $n = 6$.
Abbreviations: HG, healthy group; ATPG, ATP-alone group; TAMG, tamoxifen-alone group; ATAG, ATP + tamoxifen group; ATP, adenosine triphosphate; MDA, malondialdehyde; tGSH, total glutathione; SOD, superoxide dismutase; CAT, catalase; 8-OHdG, 8-hydroxy-2′-deoxyguanosine; df, degrees of freedom; Sig., significance (p value).
