# Supplementary material for: Protective Role of Adenosine Triphosphate Against Tamoxifen-Induced Retinal Toxicity in a Rat Model
Source: Medicina (Kaunas). 2026 Apr 19;62(4):787. doi: 10.3390/medicina62040787 (PMC13117042; doi:10.3390/medicina62040787)
Supplement: Supplementary file 1 [file medicina-62-00787-s001.zip › Table S2-R1.pdf]

Table S2. Levene's test results for homogeneity of variances of biochemical parameters in rats

	Biochemical Variables				
	**MDA**	**tGSH**	**SOD**	**CAT**	**8-OHdG**
**Levene's statistic**	4.064	7.015	6.391	0.955	0.094
**df1**	3	3	3	3	3
**df2**	20	20	20	20	20
**Sig.**	0.021	0.002	0.003	0.433	0.962

Footnotes: Levene's test revealed a violation of the homogeneity of variances assumption for MDA, tGSH, and SOD; accordingly, the Games–Howell post hoc test was applied for these variables. In contrast, for CAT and 8-OHdG, where the assumption of variance homogeneity was satisfied, Tukey's Honestly Significant Difference (HSD) test was employed.

Abbreviations: MDA, malondialdehyde; tGSH, total glutathione; SOD, superoxide dismutase; CAT, catalase; 8-OHdG, 8-hydroxy-2′-deoxyguanosine; df1, numerator degrees of freedom; df2, denominator degrees of freedom; Sig., significance (*p* value).
