# Supplementary material for: Protective Role of Adenosine Triphosphate Against Tamoxifen-Induced Retinal Toxicity in a Rat Model
Source: Medicina (Kaunas). 2026 Apr 19;62(4):787. doi: 10.3390/medicina62040787 (PMC13117042; doi:10.3390/medicina62040787)
Supplement: Supplementary file 1 [file medicina-62-00787-s001.zip › Table S3-R1.pdf]

Table S3. Assessment of the normality of retinal layer thickness measurements in rat retina using the Shapiro–Wilk test

			Retina thickness of layers			
		**Shapiro –Wilk**	**IPL**	**INL**	**ONL**	**TR**
Groups	HG	Statistic	0.986	0.935	0.938	0.981
		df	36	36	36	36
		Sig.	0.909	0.036	0.045	0.763
	ATPG	Statistic	0.984	0.945	0.963	0.963
		df	36	36	36	36
		Sig.	0.861	0.075	0.273	0.266
	TAMG	Statistic	0.947	0.968	0.974	0.964
		df	36	36	36	36
		Sig.	0.086	0.361	0.543	0.292
	ATAG	Statistic	0.955	0.924	0.963	0.987
		df	36	36	36	36
		Sig.	0.152	0.016	0.268	0.944

Footnotes: Given that Shapiro–Wilk analysis demonstrated deviations from the assumption of normality in the INL and ONL, and that Levene's test indicated variance heterogeneity, group comparisons were conducted using Welch's ANOVA, a method robust to violations of normality and homogeneity of variances; post hoc multiple comparisons were subsequently performed using the Games–Howell test. For all groups, $n = 36$.
Abbreviations: HG, healthy group; ATPG, ATP-alone group; TAMG, tamoxifen-alone group; ATAG, ATP + tamoxifen group; ATP, adenosine triphosphate; IPL, inner plexiform layer; INL, inner nuclear layer; ONL, outer nuclear layer; TR, total retina; df, degrees of freedom; Sig., significance (*p* value).
