# Supplementary material for: Protective Role of Adenosine Triphosphate Against Tamoxifen-Induced Retinal Toxicity in a Rat Model
Source: Medicina (Kaunas). 2026 Apr 19;62(4):787. doi: 10.3390/medicina62040787 (PMC13117042; doi:10.3390/medicina62040787)
Supplement: Supplementary file 1 [file medicina-62-00787-s001.zip › Table S4-R1.pdf]

Table S4. Levene's test results assessing the homogeneity of variances for retinal layer thicknesses

	Retina thickness of layers			
	**IPL**	**INL**	**ONL**	**TR**
**Levene's statistic**	3.489	2.348	9.491	8.953
**df1**	3	3	3	3
**df2**	140	140	140	140
**Sig.**	0.017	0.075	<0.001	<0.001

Footnotes: Levene's test revealed a violation of the homogeneity of variances assumption for IPL, ONL, and TR; accordingly, the Games–Howell post hoc test was applied for these variables. For INL, despite the homogeneity of variances indicated by Levene's test, deviations from normality were observed; therefore, group comparisons were conducted using Welch's ANOVA followed by the Games–Howell post hoc test.

Abbreviations: IPL, inner plexiform layer; INL, inner nuclear layer; ONL, outer nuclear layer; TR, total retina; df1, numerator degrees of freedom; df2, denominator degrees of freedom; Sig., significance (*p* value).
