# Supplementary material for: Protective Role of Adenosine Triphosphate Against Tamoxifen-Induced Retinal Toxicity in a Rat Model
Source: Medicina (Kaunas). 2026 Apr 19;62(4):787. doi: 10.3390/medicina62040787 (PMC13117042; doi:10.3390/medicina62040787)
Supplement: Supplementary file 1 [file medicina-62-00787-s001.zip › Table S5-R1.pdf]

Table S5. Post hoc multiple comparisons of p-values reflecting the effects of ATP and tamoxifen on oxidant, antioxidant, and oxidative DNA damage biomarkers in rat ocular tissue

	**Post hoc *p*-values**				
**Group comparisons**	**MDA***	**tGSH***	**SOD***	**CAT****	**8-OHdG****
HG vs. ATPG	0.740	0.717	0.654	0.223	0.437
HG vs. TAMG	<0.001	<0.001	<0.001	<0.001	<0.001
HG vs. ATAG	0.888	0.837	0.027	0.115	0.959
ATPG vs. TAMG	<0.001	<0.001	<0.001	<0.001	<0.001
ATPG vs. ATAG	0.592	0.325	0.028	0.002	0.210
TAMG vs. ATAG	<0.001	<0.001	<0.001	<0.001	<0.001
F-value	228.943[a]	494.046[a]	318.691[a]	149.910	41.970
df (df1 / df2)	3 / 10.832	3 / 10.427	3 / 10.694	3 / 20	3 / 20
p	<0.001[b]	<0.001[b]	<0.001[b]	<0.001	<0.001

Footnotes: *Welch's ANOVA was used for statistical analyses, and post hoc multiple comparisons were performed using the Games–Howell test. ** Statistical analyses were conducted using one-way ANOVA, followed by Tukey's Honestly Significant Difference (HSD) test for post hoc multiple comparisons. [a] denotes values that are asymptotically F distributed. [b] indicates *p*-values derived from Welch's ANOVA. For all groups, $n = 6$.

Abbreviations: HG, healthy group; ATPG, ATP-alone group; TAMG, tamoxifen-alone group; ATAG, ATP + tamoxifen group; ATP, adenosine triphosphate; MDA, malondialdehyde; tGSH, total glutathione; SOD, superoxide dismutase; CAT, catalase; 8-OHdG, 8-hydroxy-2′-deoxyguanosine; df, degrees of freedom; df1, numerator degrees of freedom; df2, denominator degrees of freedom.
