# Supplementary material for: Protective Role of Adenosine Triphosphate Against Tamoxifen-Induced Retinal Toxicity in a Rat Model
Source: Medicina (Kaunas). 2026 Apr 19;62(4):787. doi: 10.3390/medicina62040787 (PMC13117042; doi:10.3390/medicina62040787)
Supplement: Supplementary file 1 [file medicina-62-00787-s001.zip › Table S6-R1.pdf]

Table S6. Post hoc multiple comparisons of retinal layer thickness measurements among experimental groups following ATP and tamoxifen administration

	Post hoc *p*-values			
**Group comparisons**	**IPL**	**INL**	**ONL**	**TR**
HG vs. ATPG	0.534	0.255	0.285	0.212
HG vs. TAMG	<0.001	<0.001	<0.001	<0.001
HG vs. ATAG	0.537	<0.001	0.021	0.449
ATPG vs. TAMG	<0.001	<0.001	<0.001	<0.001
ATPG vs. ATAG	0.033	<0.001	<0.001	0.034
TAMG vs. ATAG	<0.001	<0.001	<0.001	<0.001
F-value	2902.127[a]	1229.826[a]	703.411[a]	7789.442[a]
df (df1 / df2)	3 / 76.095	3 / 76.803	3 / 75.579	3 / 74.107
p	<0.001[b]	<0.001[b]	<0.001[b]	<0.001[b]

Footnotes: All statistical analyses were performed using Welch's ANOVA, followed by the Games–Howell post hoc test for multiple comparisons. a indicates Welch's F statistics. b denotes *p*-values obtained from Welch's ANOVA. For all groups, $n = 36$.

Abbreviations: HG, healthy group; ATPG, ATP-alone group; TAMG, tamoxifen-alone group; ATAG, ATP + tamoxifen group; ATP, adenosine triphosphate; IPL, inner plexiform layer; INL, inner nuclear layer; ONL, outer nuclear layer; TR, total retina; df, degrees of freedom; df1, numerator degrees of freedom; df2, denominator degrees of freedom.
